# Supplementary material for: A Single-Institute Experience with C-ros Oncogene 1 Translocation in Non-Small Cell Lung Cancers in Taiwan
Source: Int J Mol Sci. 2022 May 21;23(10):5789. doi: 10.3390/ijms23105789 (PMC9145855; doi:10.3390/ijms23105789)
Supplement: Supplementary file 1 [file ijms-23-05789-s001.zip › ijms-1729788-supplementary.pdf]

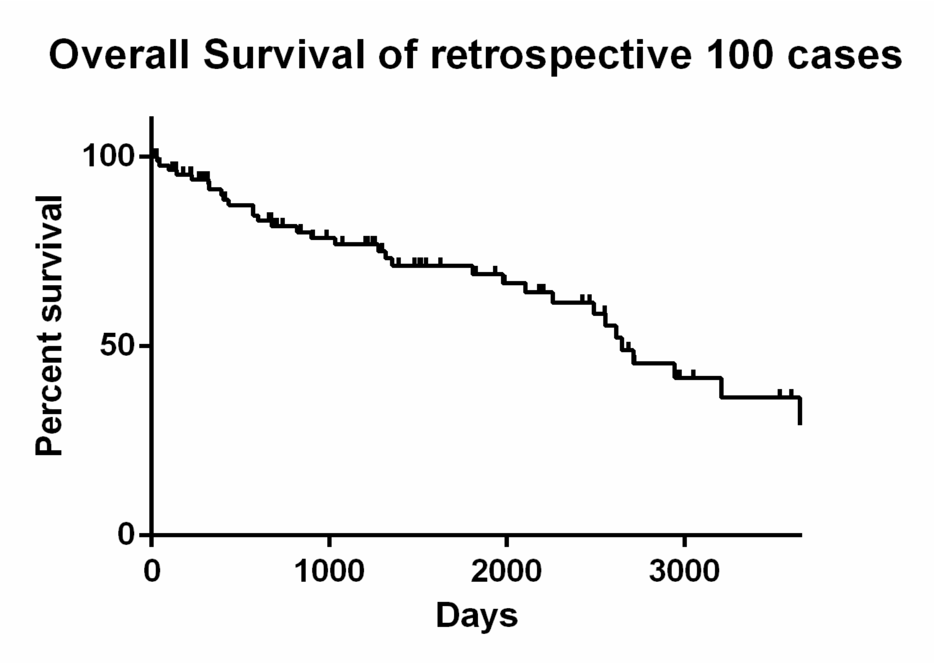

Supplementary Figure S1. Overall survival of the 100 cases in pre-crizotinib period. The overall 1-year, 3-year, 5-year and 10-year survival values of the 100 NSCLCs are 91%, 77%, 69% and 36%.
